# Compliance With Higher Education-Related Tobacco Control Law Provisions by Institutions of National Importance in India

**DOI:** 10.7759/cureus.42129

**Published:** 2023-07-19

**Authors:** Raja Singh

**Affiliations:** 1 Architecture, School of Planning and Architecture, New Delhi, IND; 2 Centre for Built Environment Policy, Information Sharing and Analysis Center, New Delhi, IND; 3 Built Environment and Public Health Fellowship Program, Tathatara Foundation, Bobbili, IND

**Keywords:** tobacco cessation, tobacco control policy, tobacco control, right to information act, india, institutes of national importance, signboards, educational institutions, anti-tobacco law, anti-smoking law

## Abstract

Introduction

India formulated an anti-tobacco and anti-smoking law in 2003 in response to its resolutions in the United Nations' bodies. This law has been detailed subsequently to make it focussed on educational institutions, which are supposed to perform on-ground action in a decentralized manner. The first step is to put up signboards prohibiting the sale of cigarettes and tobacco products within 100 yards, the second is prohibiting smoking within the campus, and the third is implementing the law related to collecting fines from the offenders as well as the presence of vendors within 100 yards. Additional information on awareness activities was also sought. The focus of this paper is on India’s premier educational institutions called the "Institutions of National Importance" by the Indian legislature. These are India’s premier public institutions which have the maximum focus of the Indian government by making them of high quality, and non-compliance with the tobacco control law in these institutions should be taken seriously.

Methods

The paper checked for compliance with the Indian Tobacco Control Law in 79 of these Institutions of National Importance. The requirement for information to be collected from institutions was first derived from the legal act and the rules. Then, the Right to Information Act of 2005, India’s transparency law, was used to file applications for information, and certified information from the institutions was collected and reported.

Results

Only 39.2% of the institutions had the requisite boards prohibiting sales within 100 yards of the institutions. The requirement of having boards prohibiting smoking fared a little better at 73.4% of institutions complying but was not universal. A total of 43% of institutions denied the information pertaining to the collection of fines, either by not providing a requisite reply or stating that this was not part of the record. The information regarding the presence of vendors was not universally supplied with 65.8% of institutions stating the same to not in record or not within the purview. With respect to the awareness activities though, 72.2% of the institutions stated to have some awareness activity for tobacco control and cessation.

Conclusion

The results show an overall weak compliance with the law. India’s health regulators and educational watchdogs must implement anti-smoking and anti-tobacco laws strictly in Indian educational institutions as this is where young people are found. Something as simple as the installation of signboards by educational institutions cannot be overlooked by them. The law must become stricter with deterrence. This must be most intense in the Institutions of National Importance which form the premier institutions in India and become the role model for other institutions in India.

## Introduction

India confers the status of Institutions of National Importance to premier public higher education institutes through legislative action by an act of parliament. This institution is defined as serving "as a pivotal player in developing highly skilled personnel within the specific region of the country" [1,2]. These institutes receive special recognition, higher autonomy, and funding from the Government of India. They are equivalent to universities in themselves and are not affiliated with any other mother university but are autonomously governed by a category-specific common council to oversee and standardize these. There are, as of 2023, 161 Institutions of National Importance in India, and these include the Indian Institutes of Technologies, National Institutes of Technology, and the All India Institutes of Medical Sciences, among other important institutes [1]. These are the crème-de-la-crème of educational institutes in India, and it is supposed that actions in these reverberate across the country. With this respect, their compliance with the law will also serve as a barometer of compliance by educational institutes in India.

India is governed as a democratic country with the rule of law. The parliament legislates and creates statutes which are called Acts. Many of these laws are created in response to the global commitments that the country makes on the level of international diplomacy and politics. Some examples are the Right for Persons with Disabilities Act 2016, which was made after a resolution of the United Nations in which India was a signatory. In the same respect, India has also enacted the Cigarettes and Other Tobacco Products (Prohibition of Advertisement and Regulation of Trade and Commerce, Production, Supply, and Distribution) Act 2003. This was done in response to the resolution in the 39th and the 43rd World Health Assembly resolutions [3]. The 43rd World Assembly resolution was all the more important as it focussed on protecting children, specifically among others. The Cigarettes and Other Tobacco Products (Prohibition of Advertisement and Regulation of Trade and Commerce, Production, Supply, and Distribution) Act 2003 or the COTPA Act 2003 hereinafter was created with the preamble to create a law which is "a comprehensive law on tobacco in the public interest and to protect the public health" and "to prohibit the consumption of cigarettes and other tobacco products which are injurious to health with a view of achieving the public health as enjoined by Article 47" of the Indian Constitution [4].

The first landmark of the COTPA Act 2003 is that it out-rightly states the below statement in Section 4.

"No person shall smoke in any public place."

This is made to include all public places with certain exceptions where designated smoking zones can be created.

In India, the Acts are the general substantive principles that are further procedurally detailed out through rules and notifications through the official gazette of India. The COTPA Act 2003 is the substantive element of the anti-smoking law in India, and this has been further detailed out to lay procedure through various rules notified thereafter by the central government. The provisions of the scope of these rules are always derived from the main law itself, which is COTPA Act 2003 in this case. For the young population, as we spoke before, the COTPA Act 2003 specifies in Section 6 as follows:

"No person shall sell, offer for sale, or permit the sale of, cigarette or any other tobacco product -

(a) To any person who is under 18 years of age, and

(b) In an area within a radius of one hundred yards of any educational institution."

In order to procedurally outline the above substantive principle, the central government notified the Cigarettes and Other Tobacco Products (Display of Boards by Educational Institutions) Rules 2009, which clearly instruct, under its Section 3, educational institutes the following [5]:

"Display of Board by Educational Institutions. (1) The owner or manager or any person in-charge of affairs of the educational institution shall display and exhibit a board at a conspicuous place outside the premises, prominently stating that sale of cigarettes and other tobacco products in an area within a radius of one hundred yards of educational institution is strictly prohibited and that it is an offense under Section 24 of the Act with fine which may extent to two hundred rupees."

The above has been made an offense under Section 24 of the COTPA Act 2003. The government has clearly defined the definition of educational institutes in Section 2(b) of the Prohibition of Sale of Cigarettes and Other Tobacco Products Around Educational Institutions Rules 2004, which"means places/centers where educational instructions are imparted according to the specific norms and include schools, colleges, and institutions of higher learning established or recognized by an appropriate authority." The distance measurement has been fine-tuned in Section 3(2) of the same as to "be measured radially starting from the outer limit of boundary wall, fence or as the case may be, of the educational institution."

The government has further notified the Prohibition of Smoking in Public Places Rules 2008, which further strengthens the anti-smoking regime. To decentralize the process of enforcement, the central government has distributed the power to book and compound the offenders under the COTPA Act 2003. The list of officers authorized to compound is provided in Schedule III of the Prohibition of Smoking in Public Places Rules, 2008. In the case of an educational institution, the Principal/Teacher /Director/Medical Superintendent/Head of the Institution is the person authorized to take action and collect the fines. This automatically means that a record of the instances of violation of the COTPA Act 2003 and its rules, along with the collection of fines, becomes the responsibility of the head of the institution. If no record is maintained, it is safe to either assume that no instances of smoking have ever taken place or no fines have ever been collected. The worst assumption, which may be true, is if the head of the institution never complied with the provisions of the said law.

The above notification, i.e., the Prohibition of Smoking in Public Places Rules 2008, also puts the responsibility on the educational institute, which falls under the category of public places, to put boards that prohibit people from smoking [6]. The verbatim provision of the law is given in Section 3(b), which is as follows:

The board as specified in Schedule II is displayed prominently as the entrance of the public place, in case there are more than one entrance at each such entrance and conspicuous place(s) inside. In case there are more than one floor, at each floor including staircase and entrance to the lift/s at each floor.

This means that the boards have to be placed at the following in a public place, including an educational institution. Firstly, at the entrance of the public place. In case of more than one entrance, at each such entrance, and secondly, on each floor, including at the staircase or lift entrance of each floor.

It is required that the board be placed at a conspicuous place where it is effortlessly visible to general visitors at first glance. The Prohibition of Smoking in Public Places Rules 2008 have also, to remove any confusion or doubt, made the specification of the required board as part of the statute.

Schedule II, with the details of the boards, is as follows:

1. The board shall be of a minimum size of 60 cm by 30 cm of white background. 2. It shall contain a circle of no less than 15 cm outer diameter with a red perimeter of no less than 3 cm wide with a picture, in the centre, of a cigarette or "beedi" with black smoke and crossed by a red hand. 3. The width of the red band across the cigarette shall equal the width of the red perimeter. 4. The board shall contain the warning "No Smoking Area - Smoking Here Is an Offence" in English or one Indian language, as applicable.

It is often stated that the COTPA Act 2003 may not have the required teeth as the maximum fine is rupees 200, which is about 2 dollars or a little more. This may not set a deterrent enough. Another route taken by the legislature has been the enactment of the Juvenile Justice (Care and Protection of Children) Act 2015, which has made selling tobacco products to a child punishable with a rigorous imprisonment of seven years and has also imposed a fine that may extend up to one lakh rupees which are about 1200 USD [7].

The question of the effectiveness of the prohibition within 100 yards of educational institutes is beyond this study. The scope is largely on whether there is compliance with the various parameters of the COTPA Act 2003 and the two rules notified under it. The major focus is on the presence of the required signboards, which create awareness and serve as a nudge to the users to not smoke or to vendors to not sell cigarettes and other tobacco products to users. Apart from this, the focus is also on whether there is an administrative measure with respect to the enforcement of warnings and fines for users who may be using cigarettes and other tobacco products within educational institutes.

Aim and objectives

This study aimed to study the compliance status of the anti-smoking/tobacco law in India’s premier higher educational institutions. There are four objectives of this study. Firstly, to study whether there is compliance in terms of the installation of the signboards that prohibit the sale of cigarettes and tobacco within 100 yards of the premier institutions in India. Secondly, to study compliance in terms of the installation of signboards prohibiting smoking within educational institutions. Thirdly, to study whether the premier educational institutions are enforcing the prohibition of smoking within the campus by imposing fines on violators of the ban on smoking. Fourthly, to know the approach of the premier educational institutes in India towards the presence of tobacco vendors within 100 yards of the educational institutions, and additionally, to study whether the premier educational institutions are undertaking any awareness activities to curb smoking and tobacco use.

Need for the study

The anti-smoking law in India, the COTPA Act 2003, is a remarkable example of anti-smoking and tobacco legislation. The landmark feature of the rules drafted after their enactment is the decentralization of the enforcement and implementation that has been done in India. This means that the on-ground work may not be fully performed directly by the government but is distributed to the organizations where the interactions take place. This means that no special force or authority as such may be responsible, but a multitude of on-ground agencies are involved. For example, in the Prohibition of Smoking in Public Places Rules 2008, the responsibility of the implementation, including the collection of fines, lies with the Head of the Institution of the educational institute. In another example, even the board that is to be placed outside of the educational institute is to be done by the educational institute and not necessarily by the municipality. This means the implementation is outsourced to the end agency.

The placement of required anti-tobacco health messaging boards, collection of fines, and awareness measures performed by educational institutions need to be checked as it reflects the performance of the law. The author has performed two similar studies in New Delhi, one based on the same methodology as this paper and the second one based on ground truthing [8,9]. In both studies, the compliance in the administrative heart of the city of the law is lackadaisical in approach.

There have been multiple studies that have looked into this issue and checked for related issues, but the uniqueness of this study lies in the number of educational institutions, and the pan-India geographic approach, which touches most, if not all, the states and union territories in India. The other factor highlighting the importance of this study is that it is performed on Institutions of National Importance, which are the premier publically financed Institutes in India. No such study in India has been performed at this scale across India in such premier institutes. Another unique element is the use of India’s transparency law for obtaining information required for this study.

This study was previously published as a preprint on medRxiv, a non-profit preprint server for health sciences, on February 21, 2023.

## Materials and methods

The following steps were performed to undertake this study. Firstly, a need for the study was established through literature. Secondly, a proforma for the information required was drafted with respect to the objectives stated in the above section. Thirdly, applications under the Right to Information Act 2005 were drafted and sent to the premier educational institutes. Fourthly, the information provided by the institute under the sign and seal through the authorized channel was collected and compiled. Fifthly, institutes where information was not provided, incomplete information was provided, or any clarification was sought were appealed under the provisions of the Right to Information. The information so derived was compiled along with the information received earlier and lastly, the information collected was reported. The educational institutions that were requested for information are summarized in Table 1. The information sought from the educational institutions is summarized in Table 2.

**Table 1:** The number of each type of educational institution that provided information under the Right to Information Act 2005. STEM: Science, Technology, Engineering, and Mathematics

S. no.	Category of educational institution	Number of institutions of this type	Remarks
1.	Indian Institutes of Management	20	These are the premier public-funded management institutions in India.
2.	Indian Institutes of Technology	22	These are the public-funded premier engineering-based educational institutes in India.
3.	National Institutes of Technology	30	These are premier engineering educational institutions in India of public nature.
4.	Indian Institutes of Science Education and Research	7	These are STEM-based fundamental science-based educational institutions in India.
Total		79	-

**Table 2:** The information requested, in brief, from Institutions of National Importance with the rationale, through the Right to Information Act 2005.

S. no.	Information requested	Why was the information requested for
1	The record of the presence, number, and locations of boards installed at a conspicuous place outside the educational institution within a radius of one hundred yards.	The law provides that all educational institutions in India put boards that prohibit the sale of cigarettes and tobacco products within 100 yards.
2	The record of the number and location of boards installed at the entrances stating "No Smoking Area - Smoking Here Is an Offense."	Apart from the boards mentioned in point 1, all public places have to have a board stating "No Smoking Area." This information is checked for compliance with the same as it prohibits the use and not the sale.
3	The information and records of the total instances of fines collected/offenses compounded/offenses recorded/warnings issued with respect to the prohibition of smoking from 2012 till date.	The collection of fines has been decentralized by law. The collection of fines is a metric in this direction.
4	The number of vendors of cigarettes and other tobacco products within a distance of 100 yards from the outer boundary of the educational institute.	Since the sale of cigarettes and tobacco products is prohibited within 100 yards, the role the educational institute plays in enforcing this is to be checked.
5	List of events/initiatives/activities/circulars/anything on record where the educational institution has taken steps to prevent the use of cigarettes and tobacco products.	Educational institutes play a role in shaping the minds of students and society. Awareness of tobacco and cigarette smoking plays a key role and is checked by this information request.

The sample size justification of the study requires first finding out the total number of Institutions of National Importance in the country. The total number of Institutions of National Importance in the country is 161 [1]. The sample for which the study has been done is 79 institutes. This means that the confidence level is 95±7.9% margin of error.

The process of collecting information through the channel of the Right to Information Act 2005 required going back and forth with the educational institutions, and in many cases, an appeal had to be made to get the maximum possible information. This study does not require ethical approval as it does not involve human subjects, and this study checks the compliance of the law with information in the public domain [10].

## Results

Out of the 79, 31 (39.2%) educational institutions had the requisite board outside of the campus in the format which prohibits the sale of cigarettes and tobacco products in an area within a radius of one hundred yards (see Section 3(1) of the Prohibition on Sale of Cigarettes and Other Tobacco Products Around Educational Institution Rules, 2004). Eight (10.1%) out of the 79 institutions had boards placed at the main entry-exit points, but they were not in the format as per mentioned in the statute. Seventeen (21.5%) confessed to having no such board placed. The remaining 23 (29.1%) educational Institutions of National Importance simply denied the information regarding the presence of the board, which prohibits the sale of cigarettes and tobacco within 100 yards of educational institutes.

With respect to the provision of a "No Smoking Area - Smoking Here Is an Offence" board in the same or similar format, 58 (73.4%) educational institutes had the requisite boards in their campus. Two (2.5%) institutions stated that they had initiated the process of installation of the boards, which was after the application for information was filed by the author. Three (3.8%) educational institutions confessed to having no boards installed. Sixteen (20.25%) educational institutions denied the information by not providing a reply.

With respect to the educational institutions penalizing people smoking on campus by issuing warnings, collecting fines, and reporting offenders, the educational institutions had the following response. Thirty-one (39.3%) educational institutions reported no incidence of smoking and therefore collected no fines. This, first of all, means that they had a mechanism to record such activities and had compliance in the first place. Fourteen (17.7%) out of the 79 institutions had taken cognizance and either issued warnings of the incidents of smoking/tobacco use within the campus and most out of the 14 institutions had collected fines, as required by the law. Thirty-four (43%) out of the 79 educational institutions denied the information by either not providing a requisite reply or by stating that this information was not part of their records.

With respect to providing information on the presence of vendors within 100 yards of the educational institutions, 24 (30.4%) out of the 79 institutions stated that there were no vendors within 100 yards of the educational institution. Three (3.8%) out of the 79 stated that there was a presence of vendors selling cigarettes and tobacco products within 100 yards of the educational institution. Fifty-two (65.8%) educational institutions denied the information or stated that there was no such record (with others stating that it was beyond their purview to have a record for the same).

The information pertaining to other steps taken by institutions to prevent the use of cigarettes and tobacco products by students in the educational institutions was asked for. Fifty-seven (72.2%) of the 79 institutions stated that they performed some kind of awareness activity. This started from the bare minimum of issuing a circular to some creating committees and other performing activities, celebrating "No Tobacco Day," etc. Some went all the way in sending complaints to local law enforcement authorities in case they saw the presence of vendors in their proximity. Five (6.3%) institutions out of the total provided no information on any activity performed, which may mean that no activity may have been performed related to awareness of smoking and tobacco use among students of the institution. Seventeen (21.5%) out of the 79 institutions did not provide the information.

## Discussion

Many studies related to the present study, but focussing on the presence of vendors of tobacco/cigarettes within 100 yards of educational institutions, have been performed in India [11-14]. This includes studies in Mohali (Punjab), Vadodara (Gujarat), and Chennai (Tamil Nadu) [15]. There was one study that was performed in Delhi but had a sample size of only 10% of schools in Delhi, and the schools were selected randomly [16]. Another Indian study was performed but was limited to the surroundings of hospital buildings as institutions, which reported a high presence of tobacco vendors [17]. But the present study is the more important and relevant for a list of reasons. This includes the large number of sample data, which is 79 educational institutions. Another important factor is that the educational institutions in this study are scattered throughout the territory of India, and this prevents any kind of regional limitation in this study. A pertinent factor making this study unique is that it has been performed using India’s transparency law, i.e., the Right to Information Act of 2005, which ensures that the information is most reliable [18]. This has been used in previous studies from other government institutions in India [19-21]. The information is reliable because it has been provided by the educational institutions themselves, that too by a senior ranking officer of the institution responsible for the actual implementation, and is provided under seal and signature of the educational institution. This nullifies the presence of any error or bias, and the data is of high integrity. On the possibility of the educational institutions providing incorrect information, it is less probable as the information has been provided in writing under law, and the officer in the institution is deemed to be aware of the consequences, which include fine and disciplinary action under the Right to Information Act of 2005 and the Indian Penal Code of 1860.

In the study, it was found that only 31% of the educational institutes had put the signages or signboards in the proper format, which prohibited the sale of cigarettes and tobacco products within 100 yards of the educational institutes. As far as the "No Smoking" boards were concerned, here too, only 58% or six in 10 Institutions of National Importance had taken the simple action of placing signages on their campus.

The effect of signboards or signages on people has a positive effect as it is an anti-tobacco health message [22]. But regardless of the effect, it is part of the legislation and forms part of the law of the land in India, and non-compliance is a violation. A study in Delhi, India also shows that compliance of the signboards also needs to be followed strictly by educational institutes [8].

The ill effects of smoking and tobacco have been reiterated by studies, but the relevance of this particular study is to the use of smoking and tobacco by young adults as it relates to higher educational institutions [23]. The use of cigarettes and tobacco at an early age affects lung health at a later age and may also serve as an impediment to quitting at an early age [24].

It has been shown in studies that the initiation of tobacco in India is at an early age [25]. This justifies the actions specified in the Indian Anti-smoking Law and its related rules, which provide for actions in educational institutes where young people are present. This is all the more necessary as the vendors, with commercial interest, usually does not ask the age of the people to whom they are selling tobacco products [26]. The prevalence of tobacco sellers within 100 yards, which may be non-permanent, "shack" based, is also prevalent [8,27].

With respect to the implementation of fines by educational institutions, this study brings to light the concept where the implementation of the smoking law has been decentralized, as the authority to collect fines and compound the offense has been distributed from law enforcement agencies to the people who are running the individual buildings or other public places. The Schedule III of the Prohibition of Smoking in Public Places Rules, 2008 gives the power to the head of educational institutes, hospital superintendents in hospitals, postmasters in post offices, station in charge in bus stands, librarians in libraries, etc. [6]. The intent is to decentralize and make it a grassroots movement. The non-compliance by India’s leading educational institutes shows a gap in the implementation of the law in India.

With respect to compliance with fines, 14 (17.7%) of the institutions actually had recorded incidences and collected fines or simply issued warnings. Another 31 simply stated that no instances were reported, and hence no fines were imposed. In the current condition in India, more studies are required and more compliance is required to actually check whether there were actually no incidences on the campus as there is considerable cigarette and tobacco use among youth in India [28]. With respect to the presence of vendors in the proximity of educational institutions, the institutions may believe that this is out of their purview. But as per "Guidelines for Tobacco Free Educational Institution" released by the Ministry of Health and Family Welfare, Government of India, the check for the presence of vendors within 100 yards and achieving absence of the same is a criterion to fetch points [29]. These points are for the self-evaluation scorecard by the educational institutions and hence this point cannot be overlooked by the educational institutions.

People may argue that the anti-smoking law in India may have less "teeth" and may not be very stringent, but with respect to the sale of tobacco products to minors, even the Indian legislature has taken the lead and made such act a serious punishment with jail time up to seven years under the juvenile justice legislation in India [7].

But there is one area where the educational institutes in this study where the results are encouraging. Fifty-seven institutions (72.2%) had actually taken some form of prevention action or the other. This may include issuing circulars, creating committees, holding counseling sessions, performing skits on tobacco-related topics, celebrating "No Tobacco Day," and undertaking inspections. This approach may be in the right direction as the students may be motivated toward tobacco control and cessation.

It is also important to highlight another critical factor in this study, and that is the use of India’s transparency law to gather the information. Around 10 institutes did not comply with the information requested fully under the Right to Information Act 2005. This means that compliance with the law is under question and an issue of concern. Overall, this study is a wake-up call for the academic institutions in India that are the ultimate torchbearers of the intention of the legislature to turn the anti-smoking and tobacco law into a reality.

## Conclusions

The study set out to find the compliance status of the anti-smoking/tobacco law in India’s premier higher educational institutions. From the study of 79 Institutions of National Importance that have been covered in this study, compliance with India’s anti-smoking/anti-tobacco law is not universal and severely lacks on many grounds. These include non-compliance with something as basic as the installation of signboards/signages within and outside of the campus prohibiting the sale/use of cigarettes and tobacco products. The five objects in the study have been tested, and in all areas, there is serious concern regarding the full implementation of the COTPA Act 2003. As a decentralized law, it is up to these educational institutes, which form an "elite group" of publically funded higher class institutes and christen themselves as Institutions of National Importance in India. The serious implementation of the tobacco control and cessation law in these Institutions of National Importance will set the right tone for other institutions in India. The educational institutions are where young people congregate and the strict implementation of tobacco control must be the utmost priority of the educational institutions by the government.
